# A data-driven approach to identifying PFAS water sampling priorities in Colorado, United States

**DOI:** 10.1038/s41370-024-00705-7

**Published:** 2024-08-01

**Authors:** Kelsey E. Barton, Peter J. Anthamatten, John L. Adgate, Lisa M. McKenzie, Anne P. Starling, Kevin Berg, Robert C. Murphy, Kristy Richardson

**Affiliations:** 1 Toxicology and Environmental Epidemiology Office, Colorado Department of Public Health & Environment, Denver, CO USA; 2 Department of Environmental & Occupational Health, Colorado School of Public Health, University of Colorado Anschutz Medical Campus, Aurora, CO USA; 3 Department of Geography and Environmental Science, University of Colorado Denver, Denver, CO USA; 4 Department of Epidemiology, Gillings School of Global Public Health, University of North Carolina, Chapel Hill, NC USA; 5 Source Water Assessment & Protection Program, Colorado Department of Public Health & Environment, Denver, CO USA

**Keywords:** Perfluorinated chemicals, Emerging contaminants, Environmental monitoring, Geospatial analyses, Vulnerable populations

## Abstract

**Background:**

Per and polyfluoroalkyl substances (PFAS), a class of environmentally and biologically persistent chemicals, have been used across many industries since the middle of the 20^th^ century. Some PFAS have been linked to adverse health effects.

**Objective:**

Our objective was to incorporate known and potential PFAS sources, physical characteristics of the environment, and existing PFAS water sampling results into a PFAS risk prediction map that may be used to develop a PFAS water sampling prioritization plan for the Colorado Department of Public Health and Environment (CDPHE).

**Methods:**

We used random forest classification to develop a predictive surface of potential groundwater contamination from two PFAS, perfluorooctane sulfonate (PFOS) and perfluorooctanoate (PFOA). The model predicted PFAS risk at locations without sampling data into one of three risk categories after being “trained” with existing PFAS water sampling data. We used prediction results, variable importance ranking, and population characteristics to develop recommendations for sampling prioritization.

**Results:**

Sensitivity and precision ranged from 58% to 90% in the final models, depending on the risk category. The model and prioritization approach identified private wells in specific census blocks, as well as schools, mobile home parks, and public water systems that rely on groundwater as priority sampling locations. We also identified data gaps including areas of the state with limited sampling and potential source types that need further investigation.

**Impact statement:**

This work uses random forest classification to predict the risk of groundwater contamination from two per- and polyfluoroalkyl substances (PFAS) across the state of Colorado, United States. We developed the prediction model using data on known and potential PFAS sources and physical characteristics of the environment, and “trained” the model using existing PFAS water sampling results. This data-driven approach identifies opportunities for PFAS water sampling prioritization as well as information gaps that, if filled, could improve model predictions. This work provides decision-makers information to effectively use limited resources towards protection of populations most susceptible to the impacts of PFAS exposure.

## Introduction

Per- and polyfluoroalkyl substances (PFAS) comprise several thousand chemicals that are ubiquitous in both the environment and human serum due to their wide use, environmental persistence, and potential for bioaccumulation [1–4]. PFAS have been manufactured since the 1940s for a variety of applications ranging from stain- and water-resistant consumer products to aqueous film forming foams (AFFF) used to fight fuel fires [1, 3–5]. PFAS are estimated to persist untransformed in water and sediment for hundreds of thousands of years [1, 3, 4]. Some longer-chain PFAS (typically defined as PFAS containing ≥6 carbons), such as perfluorooctane sulfonate (PFOS), and perfluorooctanoate (PFOA), have estimated elimination half-lives as long as 2–8 years in humans [6–9]. Exposure to certain PFAS associated with health effects including increased cholesterol, decreased infant birth weight, and increased risk of certain types of cancer [10]. Certain populations, including infants, young children, and populations disproportionately burdened by environmental pollution, may face greater risk of health impacts from PFAS exposure [11–14].

Between 2013 and 2015, the United States Environmental Protection Agency (USEPA) tested approximately 8% of U.S. public water systems for a panel of six PFAS. This effort sampled water provisions for a total of 240 million people as part of the Third Unregulated Contaminant Monitoring Rule (UCMR3) [15]. UCMR3 detected PFAS in the water of 16 million people across 33 states, including multiple water systems serving a combined 65,000 people in Fountain Valley, Colorado (El Paso County) where PFAS were detected over the 2016 USEPA health advisory (70 ng/L) [16, 17].

After the discovery of PFAS contamination in El Paso County, the Colorado Department of Public Health and Environment (CDPHE) conducted additional water testing and identified other contaminated water systems and surface water sites across the state [18–20]. In the CDPHE’s 2020 PFAS Sampling Project, all 71 surface water sites had detectable concentrations of PFAS and approximately 25% of the 397 treated drinking water systems had detectable concentrations of PFOS or PFOA [19]. While the CDPHE was able to collect data on the drinking water exposure of 75% of the population, many smaller and rural public water systems, and nearly all private wells, remain untested [19, 21].

Due to the ubiquitous nature of PFAS and the multitude of potential sources, prioritizing limited resources for water sampling efforts is challenging. Building on studies that aimed to develop similar prediction models and improved understanding of the dynamics driving PFAS in the environment [22–25], the goal of this project was to incorporate data on all known and potential PFAS sources, including factors that may impact groundwater vulnerability and PFAS transport in the environment, and existing PFAS water sampling data in Colorado into a PFAS groundwater contamination risk prediction map. Additional goals included evaluating the potential for exposures in disproportionately impacted communities and developing recommendations for a prioritization plan to inform future sampling efforts and resource allocation.

## Methods

### Data sources

This project used a variety of data sources, including PFAS water sampling results, information on known and potential PFAS sources, physical environmental characteristics, population density, and population vulnerability. Basic information on each data source can be found in Tables 1–3, with additional information included in Supplementary Tables 1, 2, and 4.

**Table 1:** PFAS groundwater sampling results compiled from 10 different sampling efforts used as the training data^a^.

Category	Data points
<5 ng/L “low risk”	705
5 ng/L ≤ × < 35 ng/L “moderate risk”	282
≥35 ng/L “high risk”	245

*PFAS* per and polyfluoroalkyl substances, *ng/L* nanograms per liter.

^a^More information about each dataset can be found in Supplementary Table 4.

**Table 2:** Potential PFAS sources, categorization approach, and sub-categories.

Source type	Categorization approach	Sub-categories
Fire stations/fire districts	Classified based on reported PFAS-containing AFFF use or storage. (*n* = 858)	No AFFF possession or use (*n* = 99)
		Possession but no use (*n* = 218)
		Possession and use (*n* = 81)
		No data (*n* = 460)
Military installations	All one category. (*n* = 13)	N/A
Airports	Classified based on AFFF certification status. (*n* = 62)	With part 139 certification that allows for AFFF testing (*n* = 14)
		Without part 139 certification that allows for AFFF testing (*n* = 48)
Landfills	All one category. (*n* = 557)	N/A
Wastewater treatment plants	All one category. (*n* = 561)	N/A
Ski resorts	All one category. (*n* = 42)	N/A
Oil and gas fire sites	All one category. (*n* = 336)	N/A
Racetracks	All one category. (*n* = 19)	N/A
Dry cleaners	All one category. (*n* = 532)	N/A
Oil and gas injection wells	All one category. (*n* = 662)	N/A
AFFF spills	All one category. (*n* = 8)	N/A
Major roadways	All one category. (*n* = 2956)	N/A
Population density	Census tract level.	N/A
Oil and gas wells	Density raster. (Kernel)	N/A
Manufacturing and industrial sites	Grouped into seven industry categories (i.e., separate feature classes) based on NAICS codes and EPA’s ranking system.^a^	Metal foil and plating. Metal foil: rank = 1, plating and platemaking: rank=3. (*n* = 203)
		Paints and varnishes. One category: rank = 3. (*n* = 38)
		Paper. Printing and paper mills: rank = 1, packaging and food containers: rank = 2. (*n* = 386)
		Refineries. Oil and petroleum: rank = 4. (*n* = 110)
		Fabrics. Waterproofing, fabric mills and finishers and leather tanning: rank = 2. (*n* = 14)
		Low impact industry. Cleaning, Service Industry, medical instruments, cosmetics: rank = 1. (*n* = 27)
		High impact industry. Lubricating oils and chemicals: rank = 3, unsupported plastics: rank = 2. (*n* = 56)
Manufacturing and Industrial Sites	Density raster. (Kernel)	All the above seven categories combined into a density raster.

All variables listed were included in the final model. Additional information about these data, including justification for inclusion as a potential PFAS source, are available in Supplemental Table 1.

*n* number of points, *PFAS* per and polyfluoroalkyl substances, *AFFF* aqueous film forming foam, *N/A* not addressed, *USEPA* United States Environmental Protection Agency.

^a^This list is based on North American Industry Classification System (NAICS) codes and compiled by USEPA. NAICS describe broad classes of industry and do not necessarily indicate which specific activities are being conducted on any given site. USEPA’s Rankings: 1: Assumed in smaller volumes or less known about process inputs; 2: PFAS assumed in after-market application; 3: PFAS assumed in multiple parts of the process and used in higher frequency or volume; 4: Direct application to environment (AFFF).

**Table 3:** Additional predictors of contamination (non-sources).

Variable name	Data type	Included in the final model?
All alluvial and unconfined aquifers, merged	Polygon, binary variable. Removed from model due to low Gini coefficient, model improved after removal.	No
Major quaternary alluvial aquifers	Polygon, binary variable.	Yes
Annual average precipitation	Matched training data to closest contour line.	Yes
Aspect	Raster, continuous.	Yes
Depth to water table	Raster, categorical. Removed from model due to low Gini coefficient, model improved after removal.	No
Elevation	Matched training data to closest contour line.	Yes
General geology	Raster, categorical.	Yes
Soil hydraulic conductivity	Raster, categorical.	Yes
Irrigated lands	Polygon, binary variable. Removed from model due to low Gini coefficient, model improved after removal.	No
Land use	Raster, categorical. Removed from model due to low Gini coefficient, model improved after removal.	No
Slope percentage	Raster, continuous.	Yes
Soil permeability class	Raster, categorical.	Yes
Water flow direction	Raster, categorical.	Yes

Additional information on these data are available in Supplementary Table 2.

### PFAS sampling results (training data)

Results from groundwater sampling from ten different efforts conducted between January, 2016 and April, 2022 were used for the training data [26]. These data included 1232 data points from public water systems, private wells, and monitoring wells. Data collected at sources, such as points from within military sites or corrective action sites, were excluded from the training data set in order to avoid introducing bias into the training data. Further, for wells sampled more than once, we used the highest value reported during the study period to generate conservative estimates with the mission of protecting public health. Maximum values represented 36 (2.9%) of the training data points. To investigate the potential impact of using maximum values rather than average values, we evaluated the classification status using average values and determined that only 8 of those 36 samples (0.06% of training data points) would be classified differently (Supplementary Table 5). More information on the training dataset can be found in Table 1 and Supplementary Table 4.

Samples that were below the limit of detection (LoD) were assigned the value of ½ the LoD. Ten datasets (Table 1 and Supplementary Table 4) were included in the training data, including data collected over multiple years for different purposes. Consequently, detection limits and the number of analyzed PFAS varied greatly throughout the training data. All datasets measured PFOS and PFOA, although some reported the summation data only.

### Known and potential PFAS sources

**Fig. 1:**
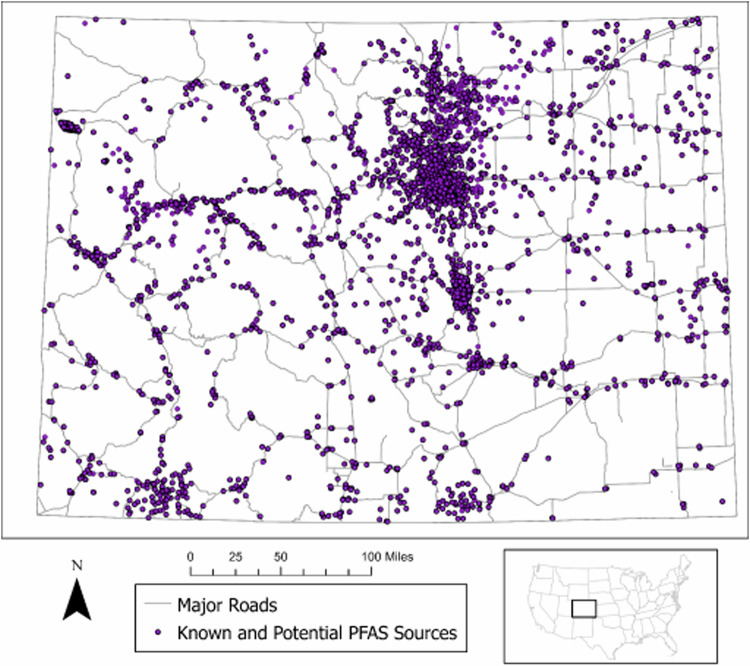
Distribution of potential and known PFAS sources in Colorado used in development of the prediction model. Does not include density features.

To distinguish known PFAS sources from unknown PFAS sources we divided each type of point source into sub-categories where possible. For example, fire stations were divided into four sub-categories based on information collected by the CDPHE in a state-wide survey of fire departments, which asked about their use of PFAS-containing firefighting foams and their participation in the fire-fighting foam registration program [27]. Not only can detailed classifications improve model performance, but this information may also be useful for identifying important point sources for predicting PFAS contamination and determining point sources that warrant further investigation. Point sources were treated as a “distance” feature in the model; specifically, the Euclidean distance from each PFAS sampling point to its nearest neighboring point source, within each specific category, was recorded. We report the distribution of known and potential PFAS point sources in Fig. 1.

We employed calculated density rasters that show where point or line features are more or less concentrated to explore the impact of varied spatial relationships for some potential source types [28]. A density raster enables analytical evaluation of potential PFAS sources that have greater uncertainty regarding the likelihood of PFAS contamination of groundwater, such as the oil and gas industry [29–31]. We calculated density rasters using the kernel density tool in *ArcPro* with default input parameters (see Table 2 and Supplementary Table 1) [32].

### Physical environmental characteristics

In addition to these point sources, we considered other explanatory variables that may predict groundwater vulnerability or shed insight into how PFAS travel through the environment. These data include elevation, soil properties, geologic features, annual average precipitation, directionality of groundwater flow, and land use (e.g., urban, agricultural, forested). Environmental characteristics were either represented as vector polygons (e.g., alluvial aquifers) or rasters (e.g., soil permeability). We present complete information on these in Table 3 and Supplementary Table 2.

### Population vulnerability

We considered population vulnerability, using Colorado’s definition of a disproportionately impacted (DI) community, as a factor in development of the sampling prioritization plan. DI communities are less likely to have equitable access to healthcare and more likely to experience a higher cumulative burden of environmental and psychosocial stressors, both of which may contribute to greater susceptibility to adverse health impacts from environmental exposures [13, 33–36]. To incorporate vulnerability into the assessment, we downloaded data from the CDPHE’s *EnviroScreen* (2022) and used the binary variable, “DI community” at the census block group level [37].

Colorado’s Environmental Justice Act defines a disproportionately impacted (DI) community as a census block group with at more than 40% low-income households, more than 40% people of color households, or more than 40% housing cost-burdened households [38]^.^ These demographic variables may impact community vulnerability to environmental exposure and disease in the following ways: low-income households face disproportionate burdens of unhealthy environmental conditions including higher exposures to air and noise pollution, water contamination, toxic waste sites and unsafe conditions of the built environment (e.g., schools and homes) [13]; due to past and present policies that perpetuate structural racism (e.g., redlining) people of color are disproportionately exposed to air pollution and heavy metals, have less access to green space, and are more likely to live near hazardous waste sites [33–35, 39, 40]; housing cost-burdened households are more likely to face material hardships including food insecurity and medical-care hardship [36].

### Statistical analysis

We employed a supervised machine learning spatial analysis technique using forest-based classification in *ArcGIS Pro 2.6.3* [41–43]. This technique creates hundreds of decision trees which are developed using a random selection of the original data (i.e., the PFAS water sampling results), referred to as the “training data” [41–43]. The model was cross-validated (in iterations of 20) with the remaining original data not used in the initial model creation. Forest-based classification uses results from each decision tree in combination to predict the outcome of an unknown sample (i.e., locations where PFAS have not yet been measured in water) [43]. We used a random sample of 75% of the data to train the model and the remaining 25% for validation [43]. The validation dataset includes the same category proportions (low/moderate/high) as the complete training dataset. The following metrics were used to determine the most appropriate set of predictors and input parameters: false positive rate, true positive rate (sensitivity), true negative rate (specificity), positive predictive value (precision), and accuracy (Supplementary Table 6) [42, 44].

To generate the most stable model possible using criteria delineated above, the model was “tuned” by adjusting tree depth and number of trees as well as the predictive variable set. The number of randomly sampled variables was set to the default which is the square root of the total number of variables. The model was run in iterations of 20 to evaluate the distribution of the variable importance box plots, which show the similarity of each variable’s importance between runs; narrower boxplots indicate greater model stability [42, 43]. Because nearly 60% of the training dataset is comprised of the “low risk” category, the “compensation for sparse categories” parameter was applied to ensure that all training data categories were equally represented in the decision tree analysis. Specifically, selecting this parameter guarantees that each category is included in each tree to create balanced models [42, 43].

**Fig. 2:**
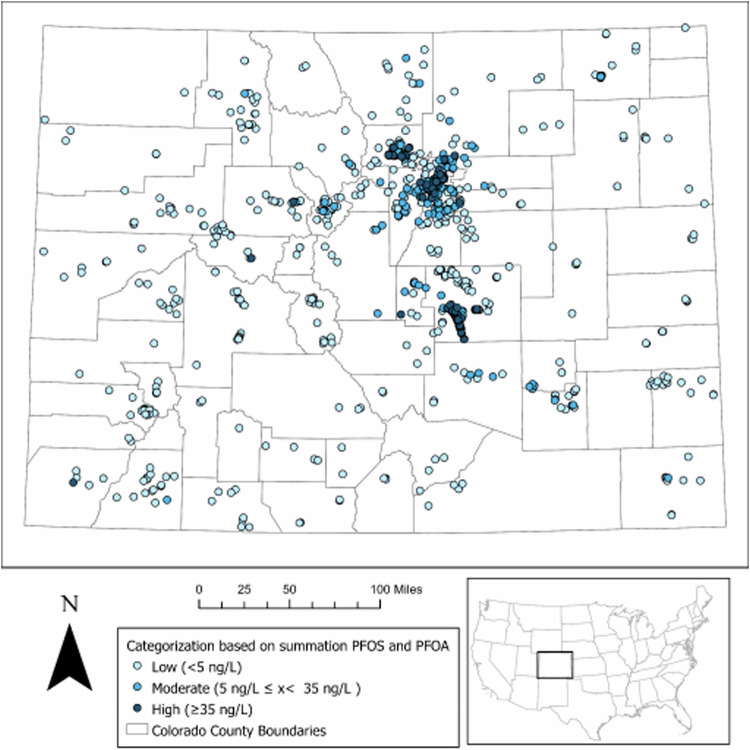
The distribution and categorization of 1232 data points with actual PFOS and PFOA used to train the model. Points are color-coded based on the categorization of the summed PFOS and PFOA concentration.  The lightest blue shows samples that are below 5 ng/L (“low risk”), the medium blue shows samples that are between 5 and 35 ng/L (“moderate risk”) and the darkest blue shows samples that are above 35 ng/L (“high risk”). Sensitive data (e.g., private wells) are off-center to protect privacy.

Once we selected the final explanatory variable set, we ran the model again using the “Train and Predict” option to generate predictions for untested locations. No data were excluded for validation in this final model run [42]. Based on the distribution of data and Colorado policies, developed from the 2016 USEPA health advisories, categories were defined as follows: 1) summation of PFOS and PFOA below 5 ng/L (“low risk”; 2) summation of PFOS and PFOA between 5 ng/L and 35 ng/L (“moderate risk”); 3) summation of PFOS and PFOA above 35 ng/L (“high risk”). The training dataset was categorized prior to training the model (Fig. 2); the model subsequently predicts a category, rather than a concentration, at unsampled locations. The number of points in each category in the training dataset are shown in Table 1. We created a one-mile point grid with *ArcPro’s* fish-net tool for the state of Colorado, and we ran the model to predict to each point in the grid across the state. Once predictions were assigned to the point grid, we transformed the grid to a surface (with continuous values ranging from 0 to 2 representing predicted risk of PFAS contamination) using an inverse distance weighting (IDW) model, a common and effective general interpolation technique [45]. To determine which variables had the most important influence on model predictions and accuracy, we assessed each variable’s Gini coefficient within the variable importance table [42].

The explanatory variables included all known and potential PFAS sources, as well as geographic features that could impact groundwater vulnerability or PFAS transport in the environment (Tables 2 and 3). Euclidean distances from each PFAS sampling location to each PFAS point source were defined by the model and assigned the term “explanatory distance features”.

### Development of a prioritization plan

Once we created the predictive map, we identified unsampled public water systems (including transient non-community systems [TNCs], non-transient non-community systems [NTNCs]), and census blocks where a high proportion of residents are private well-users. TNCs are public water systems that provide water to at least 25 people over a short period of time (e.g., gas stations and campgrounds). NTNCs are public water systems that provide water to at least 25 people for at least 6 months per year (e.g., schools and hospitals) [46]. We assigned all unsampled water systems that rely on groundwater, and private well locations determined from the Colorado Division of Water Resources dataset, a PFAS groundwater contamination risk value based on the risk surface described above. Due to the interpolation procedure, each assigned risk value was a continuous number between 0 (lowest risk) and 2 (highest risk). We developed the priority sampling list based on the potential exposure of vulnerable populations and the predicted groundwater contamination risk. Specifically, we evaluated schools with independent water systems, mobile home parks, and census blocks considered to be disproportionately impacted that were at elevated groundwater contamination risk and had higher private well density. We also considered other unsampled systems at elevated risk not covered under the Fifth Unregulated Contaminant Monitoring Rule (UCMR5) sampling [47]. Finally, we assessed data gaps and evaluated the variable importance ranking and distance to point sources to explore data and mapping needs to improve the predictive power of the model.

## Results

### Map diagnostics

Table 4 presents diagnostics for the selected model. This model yielded the best fit after adjusting input parameters and exploratory variables. Several non-source predictors were excluded from the final model, based on low Gini coefficients and lack of influence in the model’s prediction power; the excluded predictors were depth to water table, land use, the complete alluvial aquifer, and irrigated lands (see Table 3). Following validation analysis, the model performed best for the “low” and “high” risk categories with 85% sensitivity and 90% precision for “low” risk and 80% sensitivity and 71% precision for “high” risk. Sensitivity and precision for the “moderate” risk category were lower at 58% and 55%, respectively. The final model, run using the full training dataset (*n* = 1232), correctly classified 96.5% of points (Supplementary Fig. 1).

**Table 4:** Diagnostics developed from the validation data (i.e., 25% training data held out for validation) for the selected model.

Risk category	Low risk (*n*)	Medium risk (*n*)	High risk (*n*)	Sensitivity	Specificity	Positive predictive value (precision)	False positive rate	Accuracy
Low risk (*n* = 177)	150	21	6	85%	88%	90%	12%	86%
Medium risk (*n* = 71)	16	41	14	58%	86%	55%	14%	80%
High risk (*n* = 61)	0	12	49	80%	92%	71%	8%	90%

*n* number of data points.

### Variable importance

The model calculates variable importance using Gini coefficients, which rank variables based on how well each variable separates samples into classes as a means of assessing its discriminatory power [48]. Population density consistently remained the most important variable throughout model runs. Other variables that ranked in the top ten for discriminatory power included ski resorts, soil permeability class, elevation, airports (without part 139 certification for AFFF testing), airports (with part 139 certification for AFFF testing), annual average precipitation, AFFF spills, fire stations (reported possession and use) and water flow direction. While the variables listed above offered marginally more predictive power than the others (shown in Tables 2 and 3), there was not a clear pattern of potential PFAS source importance with the exception of population density, which suggests a need for additional investigation and understanding of most potential source types (Supplementary Table 3).

### Sampling prioritization: drinking water

**Fig. 3:**
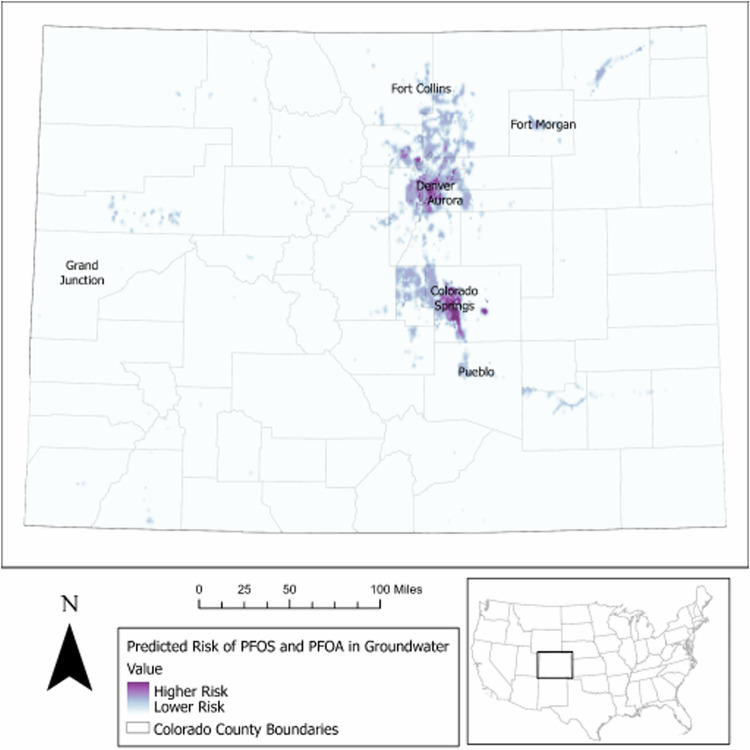
Predicted PFOS and PFOA risk in Colorado groundwater where the lightest purple indicates lowest predicted risk and the darkest purple indicates highest predicted risk (Version 1 January 2023).

We derived sampling prioritization from the predictive map (Fig. 3) alongside information on DI communities, unsampled public water systems (including very small community water systems, TNCs and NTNCs) and private wells. With this approach, we developed recommendations to target specific schools, mobile home parks, census blocks with high proportions of private-wells, and other potentially at-risk public water systems for the CDPHE’s PFAS sampling programs [19].

We identified fifteen schools and nineteen mobile home parks with unsampled drinking water systems as having potentially elevated risk of PFOS and PFOA groundwater contamination. Three of the schools and twelve of the mobile home parks are located in DI communities. We also identified over 300 public water systems at potentially elevated risk for PFOS and PFOA groundwater contamination, most of which are very small (*n* = 70) or considered TNCs (*n* = 152) and NTNCs (*n* = 32). Lastly, we identified 20 priority census blocks in DI communities each containing at least 5,000 household use or domestic use private wells according to available data.

### Sampling prioritization: source investigation

As noted above, the variable importance ranking indicated a need for additional investigation of many source types. In Colorado, the primary source types that have been investigated with targeted sampling include Department of Defense sites, and a limited number of fire stations, airports, corrective action sites, and wastewater treatment plants. Many source types included in this model have not undergone targeted sampling to evaluate occurrence of PFAS releases or contaminant plume delineations. We evaluated the distance metrics (i.e., the distance from each sampling point in the training dataset to the nearest point of the source type) for each source type to determine which source types had the least amount of PFAS sampling near them.

The distance variable (Supplementary Table 3) suggests that improving our understanding of historic and ongoing PFAS-containing AFFF use would aid in PFAS investigation and enable better mapping and characterization of PFAS contamination in Colorado. While the CDPHE has conducted surveys of fire stations on PFAS-containing AFFF use and possession, historic use of PFAS-containing AFFF is largely not recorded. Identifying past use cases and conducting representative sampling could help to further identify and reduce exposure.

## Discussion and conclusion

The purpose of this prediction map was to inform a first-round prioritization approach that targets areas of Colorado with higher risk of PFOS and PFOA contamination and determine information that could be collected to improve the predictive power of future mapping work. From 2023 to 2025, small (between 3000 and 10,000 people) and large (>10,000 people) public water systems are required to test for PFAS during implementation of UCMR 5 [47]. This leaves over 1800 public water systems (including TNCs and NTNCs) without a testing requirement in Colorado and does not include testing of private wells [49]. Further, in 2024 the USEPA released finalized maximum contaminant levels for six PFAS, including PFOS and PFOA [50, 51]. This rule requires sampling for PFAS by public water systems but will not impact private wells or TNCs. The lack of information for private well users means that PFAS risk may continue unchecked.

### Recommendations

To address these data gaps, we recommend a particular focus on smaller systems serving rural areas, mobile home parks, and schools that provide water to vulnerable or disproportionately impacted populations. We also recommend a focus on census blocks that have a high proportion of private well users and are considered DI communities.

To maintain focus on vulnerable populations, we have prioritized schools and mobile home parks for sampling and impact assessment efforts. While water systems that serve schools are considered to be NTNC systems, students and employees may spend a majority of their waking time at school, where they may consume significant amounts of drinking water. The lack of information on the temporal relationship between PFAS exposure and the onset of observable symptoms obscures the causal links. However, children are more likely to experience higher exposure per body weight than adults, and studies have found they often carry a higher PFAS body burden than adults [11, 14]. Further, many health effects associated with PFAS exposure have been shown to manifest during adolescence [11, 14, 52].

Mobile home parks often are commonly located in DI communities, providing housing for lower-income and socially vulnerable individuals. Mobile home parks often do not have the resources to provide adequate environmental services, including drinking water, storm water and wastewater drainage [53]. A nationwide study found that living in a mobile home park was negatively associated with water service reliability [54]. Further studies in California observed that mobile home parks were more likely to incur violations for health-based standards than their non-mobile home counterparts [55]. To address some of these concerns, Colorado passed a Mobile Home Park Water Quality bill in 2023 which created a water testing program, which may include testing for PFAS, for mobile home parks [56].

This work suggests that TNCs and NTNCs should be prioritized as sampling sites. While TNCs and NTNCs are often overlooked in sampling programs due to the limited exposure duration for many who utilize them, people may use these systems for their primary or secondary water source throughout the year. Key examples include employees and students who drink from water systems at churches and schools. We therefore include these system types, alongside very small public water systems (<3000) in our sampling list if they are in an area predicted to have elevated contamination risk. Care should be taken to prioritize sampling of these systems not only by predicted risk levels and DI community status, but also by assessing impacts to potentially vulnerable populations including infants and young children, and people who are pregnant or planning to become pregnant, or currently breastfeeding (e.g., evaluate age structure of community served or metrics such as proportion of people participating in the Special Supplemental Nutrition Program for Women, Infants, and Children [i.e., WIC]).

Because there are limited resources to sample private wells, resources should be focused on DI communities with a high proportion of private well users whose groundwater may be at risk of PFAS contamination. Private well owners in DI communities may not have the resources to regularly test and treat their water. CDPHE may also have resources to connect well-owners to free or reduced-cost filtration options through its emergency assistance program [19]. Therefore, a one goal of this effort was to highlight areas with high densities of private wells for targeted outreach to increase enrollment into the CDPHE’s PFAS sampling programs. Because private wells are spatially dispersed throughout Colorado [57]; increased well testing will also help to address important data gaps.

Finally, this report suggests attention be given to specific source types where limited information is available on PFAS releases. Records of PFAS use or release are absent because over decades of use these substances were not subject to regulation under various environmental regulations that monitor and regulate releases. The lack of clear patterns in variable importance for most potential PFAS source types points to a need for additional source investigation. CDPHE may explore its authority to investigate various PFAS source types. In 2018, CDPHE added PFOS and PFOA to its state hazardous constituent list. This gives CDPHE the authority to monitor for and address PFOS and PFOA at facilities subject to corrective action under the Resource Conservation and Recovery Act (RCRA). Other sources could be investigated indirectly by sampling groundwater from private wells and/or surface water likely influenced by those sources [19, 58]. At a national level, changes to Toxic Release Inventory reporting to remove the *de minimis* exemption, as well as designation of PFOS and PFOA as hazardous substances under Comprehensive Environmental Response, Compensation, and Liability Act (CERCLA), will improve accuracy of information available for future releases of PFAS; however, gaps will persist without investigation of historic or ongoing releases at potential sources [50, 59]. Improved understanding of release volumes of PFAS containing substances at each source location may also be used in the future as a way to rank and prioritize potential sources.

In future model iterations where the variable importance ranking provides better understanding of the predictive power of various PFAS sources, it will be important to evaluate the spatial structure of the data to consider the effects of spatial autocorrelation. Additionally, as we move towards more consistency in analytical detection capabilities and the number of PFAS analyzed, future work could incorporate time-trend analysis to assess changes in PFAS contamination patterns over time.

### Model strengths

Some strengths of this model include the ability to incorporate and auto-calculate spatial factors (e.g., distance to source) into the prediction model, employ numerous and varied explanatory variables, assess which factors are most predictive of PFAS contamination through the model’s variable importance function, validate many different model iterations with varied input specifications before running the final prediction model, and visually present results in a manner that facilitates understanding and decision-making. This analytical approach has been demonstrated to be effective in projects with similar objectives [22, 24, 60–62], including a recent study that evaluated the effectiveness of random forest classification in comparison to logistic regression for predicting PFAS contamination in private wells in New Hampshire [23]. The authors also found that random forest classification (performed in R rather than ArcGIS Pro), performed better than logistic regression across all five PFAS evaluated [23].

Random forest classification is a particularly effective analytical tool for this project due to its ability to develop predictive maps using many different covariates without an assumed linear relationship with the outcome variable, as is the case with the data used here. Further, random forest classification has no assumptions about normalcy, yielding a model that can effectively handle non-parametric, ordinal, and categorical data [41].

### Model limitations

Limitations largely stem from data sources themselves, including high LoDs in some portion of the samples, preferential sampling of PFAS at contaminated sites and across Colorado’s Front Range, inconsistency in the number of PFAS analyzed per site, and limited knowledge of occurrence, magnitude, and duration of PFAS releases at most point sources.

With respect to the range of LoDs, there are 51 samples (4% of training data) included in the training dataset which are non-detect but classified in the moderate category rather than the low category due to high LoDs and the substitution method we employed in this work. It is possible that this biases the model predictions high near some sources. For example, one dataset collected in Frisco, Colorado has LoDs of 20 ng/L and 10 ng/L for PFOA and PFOS, respectively. Eleven samples were collected in this area because of nearby sampling results indicating PFAS contamination. All eleven samples in this area came back below detection, but in the training dataset are classified as moderate risk. We do not have extensive data in this area, which is near a ski resort. This could bias the model towards the ski resort source. More information needs to be collected in areas like this to verify results for subsequent model iterations.

On the other hand, 27 of the 51 high LoD samples were collected in El Paso County in relation to investigations of AFFF release from a military base. We have hundreds of samples from this area indicating widespread contamination. The LoDs for these samples are higher compared to other datasets because they were collected in the earlier years of PFAS investigation (2016 and 2018). Given known contamination in this area, it is not as likely that the classification for these 27 samples biases model predictions. Finally, changing LoDs and differences between regulatory quantitation limits and laboratory methods have complicated our ability to assess risk in the past. As we continue to collect data via standardized methods for PFAS with increasingly lower LoDs, we will improve risk classification models and decision-making.

Random forest classification is more effective at handling preferential sampling than alternative methods [61]. It is important to ensure the training dataset (75% of the PFAS sampling results) represents adequate geographic spread and has similar proportions in each category as the full dataset. To account for differences in PFAS results data (number of PFAS analyzed and differences in detection limits) we decided to move forward with a simple aggregation of PFOS and PFOA. While this limits our ability to predict the full spectrum of PFAS contributing to contamination, it enables us to tease out point sources or geographic features that contribute to commonly detected PFAS. While there are some differences in the sources of PFOS and PFOA, there are also many similarities and these two PFAS are often found together. In 92% of the training data sampling points the detection status of PFOS and PFOA are the same. In other words, only in 8% of the samples was PFOA detected and PFOS not detected or vice versa. As Colorado continues to collect data with lower LoDs and an expanded suite of PFAS we would work to rerun this model for separate PFAS types to better understand source signatures, fate, and transport in Colorado. Finally, while a continuous-scale analysis may be more informative for identifying water systems at highest relative risk, the current data do not suggest benefits of performing regression analysis with continuous values over the type of classification we employed. The merits of classification are further supported in a review of machine learning models to predict potential groundwater contamination, which determined that models generally performed better with classification than regression [63]. While the data used in this work was better suited for classification analysis, whether to run classification or regression is dependent on the type and extent of available data.

Additional information on source types could be useful for better refining the model in the future. For example, landfills likely have a different risk of PFAS contamination based on factors such as age of waste and how the landfill is constructed [64–66]. Further, the depth to water table data set could be improved to better approximate well depth across Colorado and potentially improve its predictive capability. We do not have or know of a reliable method for evaluating well depths at the statewide scale. The high variability of aquifer types and characteristics across Colorado (fracture flow, alluvial, confined, unconfined, etc.), fluctuating and depleted aquifers without water-level monitoring data, and significant variability in transmissivity, additionally introduces complexity that becomes a challenge at the statewide scale. Moving forward, we have interest in undertaking a similar spatial/modeling exercise at more localized or regional scales that incorporate well depth or “uppermost groundwater aquifer” depth in heavily studied and understood aquifer systems with time-series monitoring data of water table depth. Finally, information on some PFAS source types and some potentially useful environmental predictors (e.g., groundwater age) [67], was not available for inclusion in the model.

### Conclusions and future work

This map represents the first iteration of this work, which we will develop further as new data become available. The primary goal of this modeling effort was to identify data gaps and drive prioritization for subsequent rounds of PFAS sampling. With its framework in place, the CDPHE will re-run this model with larger and more comprehensive training data on an annual basis. Additional data with more consistent and lower LoDs will allow for an assessment that is more refined and improve model predictions. Further, a better understanding of releases from potential PFAS sources, along with targeted sampling in higher risk areas, will assist resource allocation efforts and improve our big-picture understanding of who is at greatest risk for PFAS exposure and health effects in Colorado.

## Supplementary information

aj-checklist

Supplemental Figure 1 Caption

Supplemental Figure 1

Supplemental Tables

## Data Availability

Data sources used in this work are described in detail in the supplemental material and on the Colorado Department of Public Health and Environment’s PFAS mapping webpage: https://cdphe.colorado.gov/pfas-mapping.
