# Supplementary material for: A data-driven approach to identifying PFAS water sampling priorities in Colorado, United States
Source: J Expo Sci Environ Epidemiol. 2024 Aug 1;35(3):414–24. doi: 10.1038/s41370-024-00705-7 (PMC12069103; doi:10.1038/s41370-024-00705-7)
Supplement: Supplementary file 2 — Supplemental Figure 1 Caption [file 41370_2024_705_MOESM2_ESM.docx]

**Supplemental Figure 1.** The classification status of the 1,232 data points with actual PFOS and PFOA used to train the model. A green point indicates that the sample was correctly classified into the low, medium or high category, respectively. A black point indicates that the sample was mis-classified (for example, the model predicted it fell into the “high” category but it was actually the “low” category). Over 96% of the training data was classified correctly in the selected model.
