# Supplementary figures and images for: A data-driven approach to identifying PFAS water sampling priorities in Colorado, United States
Source: J Expo Sci Environ Epidemiol. 2024 Aug 1;35(3):414–24. doi: 10.1038/s41370-024-00705-7 (PMC12069103; doi:10.1038/s41370-024-00705-7)

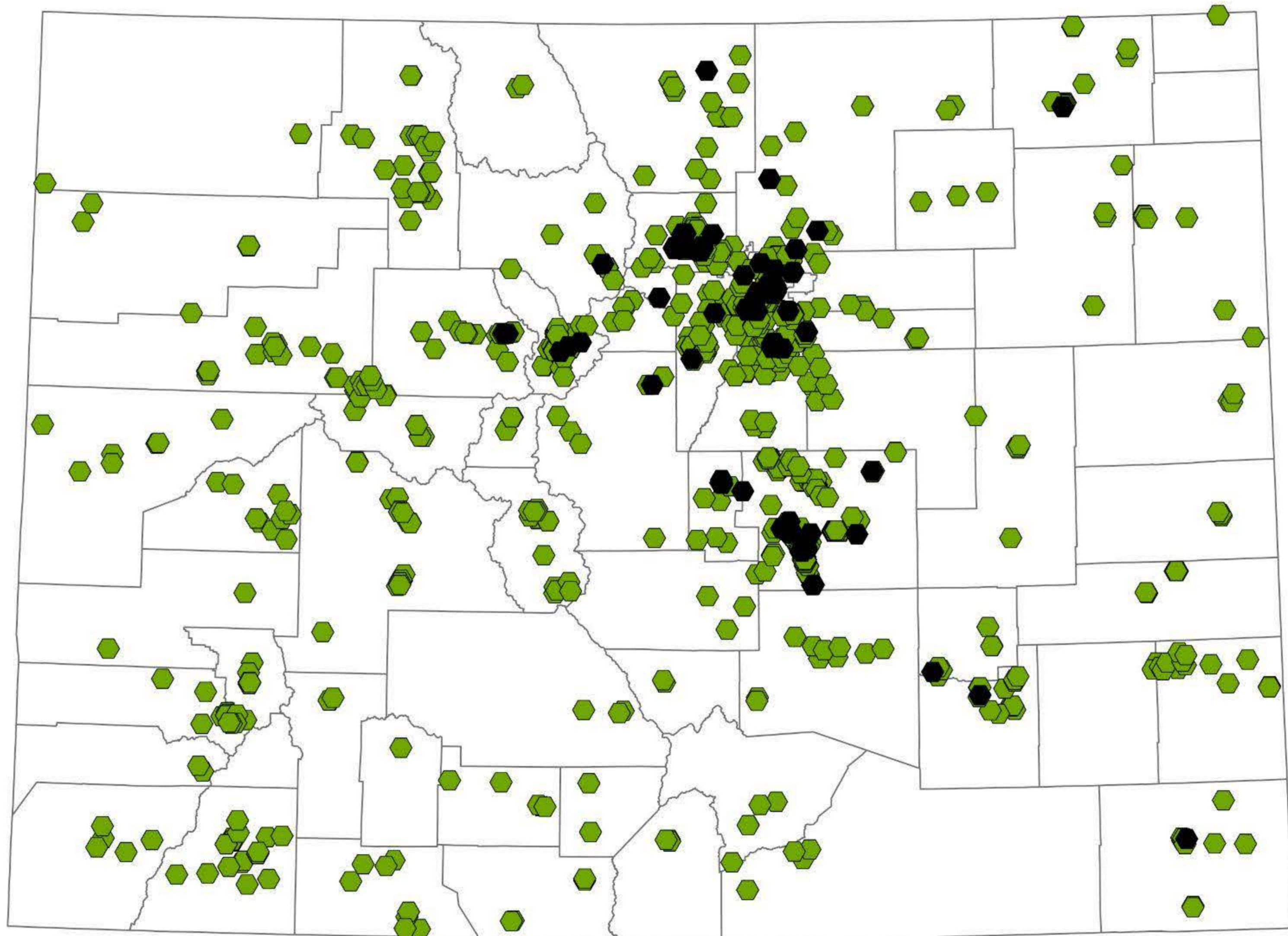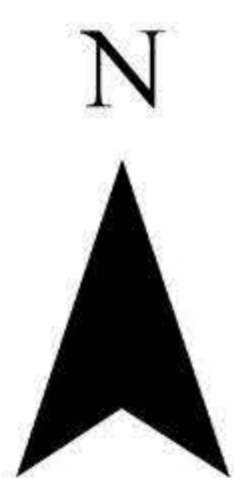

0 25 50 100 Miles

Correctly Classified

● No

● Yes

□ Colorado County Boundaries

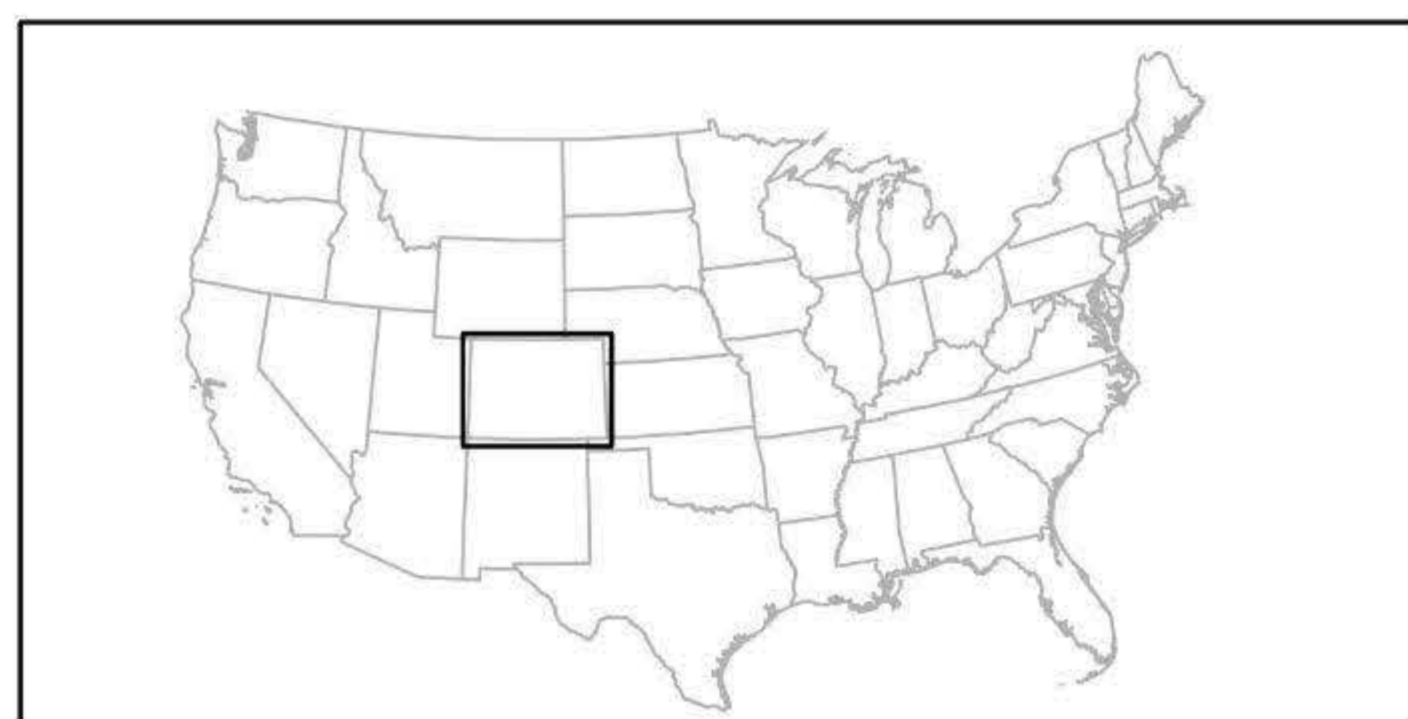

Supplement: Supplementary file 3 — Supplemental Figure 1 [file 41370_2024_705_MOESM3_ESM.pdf]
